# Utilizing Variants Identified with Multiple Genome-Wide Association Study Methods Optimizes Genomic Selection for Growth Traits in Pigs

**DOI:** 10.3390/ani13040722

**Published:** 2023-02-17

**Authors:** Ruifeng Zhang, Yi Zhang, Tongni Liu, Bo Jiang, Zhenyang Li, Youping Qu, Yaosheng Chen, Zhengcao Li

**Affiliations:** 1 State Key Laboratory of Biocontrol, School of Life Sciences, Sun Yat-Sen University, Guangzhou 510006, China; 2 Institute of Neuroscience, Panzhihua University, Panzhihua 617000, China; 3 Genetic Data Center, Faculty of Forestry, University of British Columbia, Vancouver, BC V6T 1Z4, Canada; 4 Guangdong IPIG Technology Co., Ltd., Guangzhou 510006, China

**Keywords:** GWAS, genomic selection, growth traits, pigs

## Abstract

**Simple Summary:**

The accurate prediction of growth traits in genomic selection (GS) is essential for pig breeding. Here, we performed GS using variants identified with three genome-wide association study methods on four growth-related traits in Yorkshire and Landrace pigs. A total of 1485 loci related to these traits and 24 candidate genes were mapped. Compared with using 60K SNP-chip data, GS with the pre-selected variants significantly improved prediction accuracies by 4 to 46% in genomic best linear unbiased prediction (GBLUP) models, and 5 to 27% in a two-kernel based GBLUP model for the four traits.

**Abstract:**

Improving the prediction accuracies of economically important traits in genomic selection (GS) is a main objective for researchers and breeders in the livestock industry. This study aims at utilizing potentially functional SNPs and QTLs identified with various genome-wide association study (GWAS) models in GS of pig growth traits. We used three well-established GWAS methods, including the mixed linear model, Bayesian model and meta-analysis, as well as 60K SNP-chip and whole genome sequence (WGS) data from 1734 Yorkshire and 1123 Landrace pigs to detect SNPs related to four growth traits: average daily gain, backfat thickness, body weight and birth weight. A total of 1485 significant loci and 24 candidate genes which are involved in skeletal muscle development, fatty deposition, lipid metabolism and insulin resistance were identified. Compared with using all SNP-chip data, GS with the pre-selected functional SNPs in the standard genomic best linear unbiased prediction (GBLUP), and a two-kernel based GBLUP model yielded average gains in accuracy by 4 to 46% (from 0.19 ± 0.07 to 0.56 ± 0.07) and 5 to 27% (from 0.16 ± 0.06 to 0.57 ± 0.05) for the four traits, respectively, suggesting that the prioritization of preselected functional markers in GS models had the potential to improve prediction accuracies for certain traits in livestock breeding.

## 1. Introduction

Genomic selection (GS) has been a routine method for genetic evaluation in animal breeding schemes [1]. It can increase the rate of genetic improvement, by reducing generation interval and providing higher accuracy of estimated breeding values. Due to the reduced cost of SNP chips and genome sequencing [2], the continuous escalation of the density of marker panels is generating both theoretical and implementation problems in breeding practice. Although improvements on prediction accuracies for some traits were occasionally observed by increasing the density of marker panels [3,4,5], in most scenarios the increase of marker density did not transform into an improved prediction accuracy, especially with a small population size. Theoretically, increasing the number of SNPs in a linear model may magnify collinearity and reduce the effects of causal variants, which will hamper the ability to prioritize relevant variants [6]. Recent studies reported that the prediction accuracy was increased by using only a limited number of SNPs associated with specific traits [7,8,9], in comparison to using low or moderate SNP-chip. In addition, SNPs around genes or potential causal variants can be preselected and integrated into genomic prediction [10]. A study highlighted that using only SNPs within quantitative trait loci (QTLs) showed a higher accuracy of the prediction of reproductive traits in two pig breeds [11]. A similar result was also observed for the prediction of residual feed intake in Duroc pigs, in which the predictive ability increased when only utilizing SNPs located in genes [12]. Moreover, combining pre-selected markers from QTLs with 50k SNP panels could result in a better prediction performance of production traits in Nordic Holstein cattle [13].

Genome-wide association analysis (GWAS) is a powerful tool to identify potential causal mutations associated with traits of interest [14,15]. It can be implemented with various statistical models, such as a mixed linear model and Bayesian model [16,17]. The mixed linear model takes one SNP into the regression model every time to analyze the relationship between marker and target traits. Significant signals might fall into a broad-range genomic region owing to the strong linkage disequilibrium between SNPs. However, the interaction of markers from an underlying QTL would be ignored [18,19]. As an alternative, Bayesian methods simultaneously fit multiple SNPs effects, mapping the genomic region on the complex trait and taking LD effect into account [20]. Performing GWAS independently on multiple populations may reduce the false positive rate and increase statistical power to find true causal mutations. However, combining summary statistics from these independent GWAS studies using meta-analysis can increase the power to find effects that are homogeneous across populations, and can elucidate between-population heterogeneity [21]. This method has gained popularity for the discovery of new candidate loci and for improving mapping precision using data from multi-breed populations in the field of livestock breeding [22,23].

Pigs are a primary source of animal protein for humans [24]. Improving pork production has always been a never-ending goal for livestock breeding. Growth-related traits such as average daily gain (ADG), backfat thickness (BFT), body weight (BW) and birth weight (BTHWT) are commonly treated as selection indices in breeding programs [25,26]. Although the genetic architecture of growth traits is largely polygenic in nature, which may be affected by a substantial number of variants with small effects, significant variants with large effects have also been discovered [14,27,28]. In this study, we use three GWAS methods including mixed linear model, Bayesian analysis and meta-analysis, with 60K SNP-chip data and whole genome sequence data from 1734 Yorkshire and 1123 Landrace pigs, to detect as many potential causal mutations as possible and assess whether utilizing these pre-selected SNPs in GS can improve the prediction accuracies of growth traits in pigs.

## 2. Materials and Methods

### 2.1. Phenotype Data

In this study, a total of 1734 Yorkshire and 1123 Landrace pigs were sampled from the nucleus farm of Guangdong IPIG Technology Co., Ltd. (Guangzhou, Guangdong, China). The 153 male and 2704 female pigs of two populations were born between 2015 and 2021. All fattening pigs were reared under uniform feeding conditions and consistent management. Phenotypic observations included 4 growth-related traits: average daily gain (ADG kg/d), backfat thickness (BFT mm), birth weight (BTHWT kg) and body weight (BW kg). When the body weight of pigs reached approximately 115 kg, backfat thickness and body weight were recorded in the test station. Backfat thickness was measured by ultrasound between the 10th and 11th rib in the live pigs. To standardize the phenotypic records, BFT and ADG were both adjusted to 100 kg and calculated by corrected equations [29]. The equation for corrected 100 kg BFT was shown below:

$$BFT\;\left(\mathrm{mm}\right)=Measured\;BFT\times \frac{A}{A+B\times \left(Measured\;body\;weight-100\;\mathrm{kg}\right)}$$

where the values of A and B for sire and dam were shown as follows:

Sire:A=13.47; B=0.1115Dam:A=15.65; B=0.156

The ADG was adjusted to 100 kg by the formula:

$$ADG\;\left(\mathrm{kg}/\mathrm{day}\right)=\frac{100\;\mathrm{kg}}{AGE}$$

where age to 100 kg (AGE) was calculated with the following formula:

$$AGE\;\left(day\right)=Measured\;age-\frac{Measured\;body\;weight-100\;\mathrm{kg}}{CF}$$

where correction factors (CF) were as follows:

$$Sire:CF=1.826\times \frac{Measured\;body\;weight}{Measured\;age}\;Dam:CF=1.715\times \frac{Measured\;body\;weight}{Measured\;age}$$

Piglets were weighed within 24 h after the delivery of each individual to measure and record the birth weight. In Yorkshire and Landrace pigs, the numbers of phenotypic records for ADG, BFT and BW were 1734 and 1123, and for BTHWT were 887 and 405, respectively. Animals with missing records were excluded from the following analysis.

### 2.2. Genotype Data

Ear tissues collected from target swine were used for genomic DNA extraction with a standard phenol/chloroform method. The A260/A280 ratio of all DNA samples was qualified by electrophoresis and a light absorption. The samples with ratio in the range of 1.8–2.0 were diluted to a final concentration of 50 ng/μL. Genotyping was conducting with the KPS Porcine 60K SNP Chip (Beijing Compass Biotechnology Co., Ltd., Beijing, China), which contains 57,466 genome-wide SNPs across 18 autosomes and sex chromosomes. SNPs with call rate less than 95% and the minor allele frequency less than 1% were filtered out using PLINK [30]. Individuals with more than 5% missing genotype were removed. Unmapped or sex-chromosome located SNPs were also excluded. The quality controls for the two populations were implemented under the same criteria, with 44,119 and 44,142 SNPs remaining for Yorkshire and Landrace pigs, respectively. In meta-analysis of GWAS, a union set of SNP-chip from two populations that passed the same quality control procedure mentioned above were later used.

### 2.3. Imputation of SNP Chips

To obtain high density SNP data, genotype phasing and imputation from 60K SNP to whole genome sequencing (WGS) data were performed by Beagle using two reference panels [31], including WGS data of 19 Yorkshire pigs and 12 Landrace pigs from NCBI Sequence Read Archive (SRA, http://www.ncbi.nlm.nih.gov/sra/ accessed on 1 July 2020) and European Nucleotide Archive (ENA, https://www.ebi.ac.uk/ena accessed on 3 July 2020) under project PRJNA260763 (Table S5). SNPs calling was performed under the following pipeline. The raw reads were trimmed by filtering adapter and low-quality bases using Trimmomatic (version 0.40) [32]. Clean reads were aligned to the pig reference genome *Sus Scrofa* (v11.1.104) by BWA, and then the aligned reads were used to detect short variants [33]. The raw SNPs were generated by employing GATK under the instruction of GATK best practice online documentation [34,35]. The GATK VariantFiltration parameter was set as “QUAL < 30.0 || QD < 2.0 || FS > 60.0 || MQ < 40.0 || SOR > 4.0 || ReadPosRankSum < −8.0 || MQRankSum < −12.5”to filter raw SNPs, followed by a further quality control step with the “--maf 0.05 --min-alleles 2 --max-alleles 2 --hwe 1e-6 --min-meanDP 5 --max-missing 0.9” option in Vcftools [36]. After the filtration, the autosome-only VCF files which contained 9,075,894 and 11,268,962 highly confident SNPs for Yorkshire and Landrace pigs, respectively, were used as reference panels of corresponding populations for genotype-imputation from the 60K SNP panel to WGS data. The average imputation accuracy based on Beagle *R*^2^ was 0.71 and 0.69 for the two populations. The imputed SNPs with minor allele frequency (MAF) < 0.01 and Hardy–Weinberg Equilibrium < 1 × 10^−6^ were discarded. Finally, the remaining 1,468,003 and 2,221,629 SNPs for Yorkshire and Landrace pigs were available for the following analyses.

### 2.4. Statistical Models for GWAS

We used three statistic models including the mixed linear model, Bayesian model, and meta-analysis for GWAS, including (1) linear model based on 60K chip data (CL_GWAS); (2) linear model based on imputed WGS data (IL_GWAS); (3) Bayesian model based on 60K chip data (CB_GWAS); and (4) meta-analysis based on 60K chip data.

#### 2.4.1. Mixed Linear Model

We performed CL_GWAS and IL_GWAS by fitting a univariate Mixed Linear Model (MLM) in GEMMA [37]:

(1) y=Wα+Xβ+u+ε

where y denotes the vector of phenotypic values; W is the incident matrices of multiple covariates, comprised of sex, days to measure and top five eigenvectors of PCA which were generated by GCTA tool [38]; α denotes the vector of corresponding coefficients including the intercept; X is the vector of SNP genotypes; β denotes the marker effect; $u\;\sim \;{\mathrm{MVN}}_{n}\left(0,\;{\lambda \tau }^{-1}K\right)$ and $\varepsilon \;\sim \;{\mathrm{MVN}}_{n}\left(0,\tau^{-1}{Ι}_{n}\right)$ represent the vector of random effects and random residuals, respectively; $\tau^{-1}$ is the variance of the residual errors; λ is the ratio of variance components of random effects to random residuals; K is a genomic relatedness matrix; ${Ι}_{n}$ is an n × n identity matrix; and ${\mathrm{MVN}}_{n}$ denotes the multivariare normal distribution with n-dimension. Bonferroni correction was used to determine the genome-wide and suggestive significance threshold values [39]. The estimated significant levels were, respectively, set as 1.13 × 10^−6^ and 2.25 × 10^−5^ for CL_GWAS, and 3.40 × 10^−8^ and 6.81 × 10^−7^ for imputed data-based GWAS.

#### 2.4.2. Meta-Analysis

Combining the CL_GWAS results from two breeds by the same trait, a meta-analysis using an inverse variance weighting method was implemented by METAL [40], in which *p* values and effects of common SNPs were used as input data. Bonferroni correction threshold for meta-analysis was identical to CL_GWAS.

#### 2.4.3. Bayesian Model

Bayes C model was implemented by GenSel, the prior assumption of which was that most markers were set as zero effect, with only a few SNPs explaining large variances [41]. Prior variance components of genetic effects and residuals were obtained using AIREMLF90 from BLUPF90 [42,43]. Gibbs-sampling chains for 50,000 iterations were run, and the first 40,000 burn-in iterations were discarded. The probFixed parameter of π was set to 0.999, which meant that one of 1000 SNPs were taken into the Bayesian analysis iterations and calculated with the given none-zero variance. The windowsBV option in GenSel was set to 1 Mb, which means that every consecutive 1 Mb SNPs region that followed the physical map order across the genome was divided into a genomic window. The percentage of genome-wide genetic variance explained by windows was utilized to identify the informative regions. Windows with the proportion of genomic variance (GV) greater than 1% were considered as significant regions, which were then taken to the downstream analyses. Genetic variances of SNP effects were estimated with the same model as in the single-marker GWAS.

### 2.5. Candidate Genes Annotation

*Sus scrofa* reference genome (version 11.1) which served as the gene location map was downloaded from Ensembl database [44]. Genes that contained or were located nearby significant SNPs which were identified using three single-marker approaches (CL_GWAS, IL_GWAS and meta-analyses) were considered as candidate genes associated with growth traits. In addition, genes which overlapped significant genomic regions (1 Mb) that explained more than 1% genetic variance proportion from CB_GWAS were also considered to be candidate genes. These genes were annotated with KOBAS 2.0 [45]. The annotated KEGG pathways and GO biological processes were confirmed under Fisher’s exact test with a significant threshold of *p* < 0.05.

### 2.6. Statistical Models for GS

#### 2.6.1. GBLUP

The model of benchmark best linear unbiased prediction (GBLUP) is [46]:

(2) y=Wα+Xg+ε

where y is a vector of phenotypic values; α denotes the vector of fixed effects; $g\sim N\left(0,G\sigma_{g}^{2}\right)$ is a vector of genomic estimated breeding value (GEBV); $\varepsilon \sim N\left(0,I\sigma_{e}^{2}\right)$ represents the vector of residual effects; W and X are incident matrices for α and g. Genomic relationship matrix G was calculated as $G=\frac{ZZ^{\prime }}{2\sum p_{i}\left(1-p_{i}\right)}$, where $p_{i}$ and Z represent MAF of markers and the adjusted MAF matrix, respectively. The G was constructed by different datasets of SNP markers depending on different strategies used [47].

#### 2.6.2. Two-Kernel Based GBLUP

The model of two-kernel based GBLUP is:

(3) $$y=W\alpha +X_{1}g_{1}+X_{2}g_{2}+\varepsilon$$

where $g_{1}\sim N\left(0,G_{1}\sigma_{g_{1}}^{2}\right)$ represents the matrix of GEBV_1_ estimated with $G_{1}$ constructed by all significant SNPs resulting from CL_GWAS, CB_GWAS IL_GWAS, and meta-analysis; $g_{2}\sim N\left(0,G_{2}\sigma_{g_{2}}^{2}\right)$ represents the matrix of GEBV_2_ estimated with $G_{2}$ constructed by the remaining SNPs in 60K SNP-chip. The sum of GEBV_1_ and GEBV_2_ were GEBV for the method.

### 2.7. Evaluation of the Accuracy of GS

Prediction accuracy (GPA) was calculated as:

(4) $$GPA=\frac{r\left(GEBV,y^{*}\right)}{\sqrt{h^{2}}}$$

where $r\left(GEBV,y^{*}\right)$ represents the Pearson’s correlation between GEBV and phenotypes corrected by fixed effects ($y^{*}$); $h^{2}$ stands for heritability of trait. All models above were implemented by BLUPF90 [43]. The predictive abilities of the defined models were measured with 20 replications of a 5-fold random cross-validation [47]. The average (SD) accuracies were presented as the results across replications.

## 3. Results and Discussion

### 3.1. Phenotypic Statistics and Heritability Estimation

Phenotypic summary statistics and heritability based on the 60K SNP-chip for four traits across two breeds are listed in Table 1. The coefficients of variation (CV) were in different ranges from 7.95% to 30.12% and 8.05% to 33.47%, which indicated that two breeds were in different variation levels. The results showed that ADG and BFT were moderately heritable for Yorkshire and Landrace, 0.44, 0.43 and 0.44, 0.40, which were similar to previous studies [14,48]. Heritability estimates of BW were also moderate, with 0.42 and 0.38 for two breeds. However, the additive genetic variance accounted for a small proportion of phenotypic inheritance in BTHWT. The heritability of Yorkshire was 0.16, consistent with the findings of [49], and the estimate of Landrace was 0.09 higher. As a low heritable trait, a great breeding improvement of BTHWT would be made by implementing marker-assisted selection or genomic selection.

### 3.2. Population Structure Analysis

**Figure 1:**
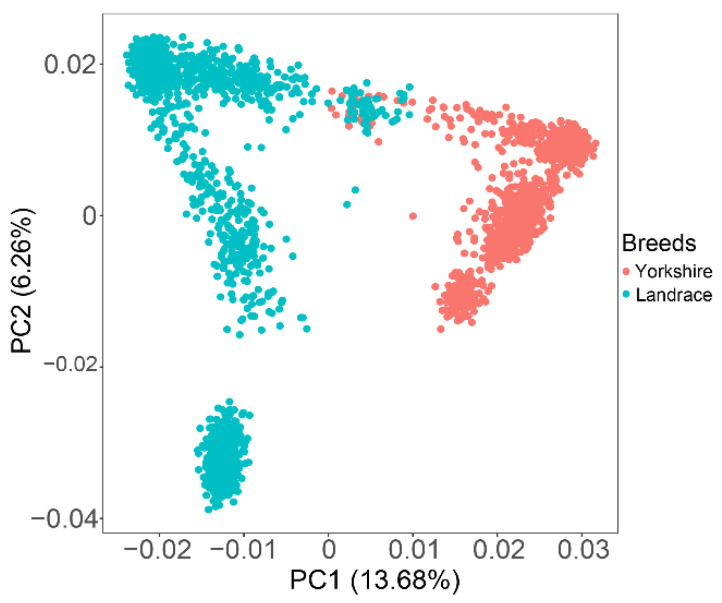
Principal component analysis shows the population structure between Landrace and Yorkshire pigs. Top two principal components: PC1 = 13.68%; PC2 = 6.26%.

Principal component analysis (PCA) was performed using a 60K SNP panel. Almost all individuals were divided into two clear clusters representing two different genetic backgrounds of target animals, with only a few individuals overlapping (Figure 1). The first two principal components explained 13.68% and 6.26% of the total variations. The top five eigenvectors were added in the GWAS models as covariates to correct for existing population stratification.

### 3.3. GWAS Based on Mixed Linear Model and Bayesian Model

**Figure 2:**
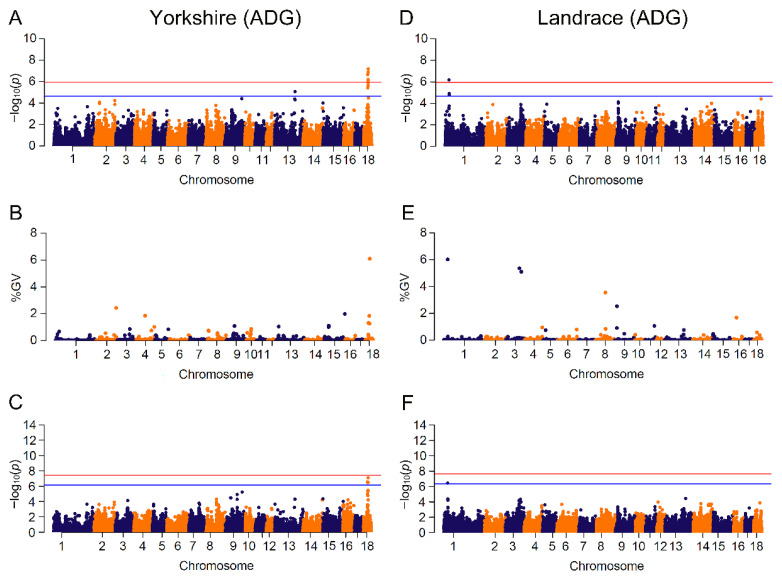
Manhattan plots for ADG (average daily gain) by breed in different GWAS approaches. The red and blue lines in plots represent genome-wide and suggestive significance thresholds, respectively. Results in Yorkshire population for: (**A**) CL_GWAS; (**B**) CB_GWAS; (**C**) IL_GWAS. Results in Landrace population for: (**D**) CL_GWAS; (**E**) CB_GWAS; (**F**) IL_GWAS. %GV denotes the proportion of genomic variance explained by the region identified.

**Figure 3:**
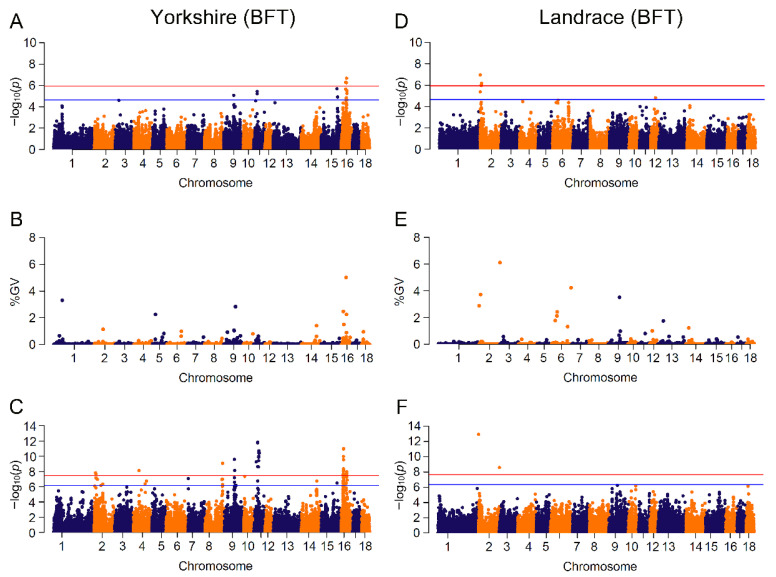
Manhattan plots for BFT (backfat thickness) by breed in different GWAS approaches. The red and blue lines in plots represent genome-wide and suggestive significance thresholds, respectively. Results in Yorkshire population for: (**A**) CL_GWAS; (**B**) CB_GWAS; (**C**) IL_GWAS. Results in Landrace population for: (**D**) CL_GWAS; (**E**) CB_GWAS; (**F**) IL_GWAS. %GV denotes the proportion of genomic variance explained by the region identified.

Results of significant genome-wide associations for four growth traits were shown in Figure 2 and Figure 3 and Supplementary Figures S1 to S4. The number of identified SNPs for CL_GWAS and IL_GWAS were 48 and 142 (Tables S1 and S3), along with 44 genomic regions that explained more than 1% of the genetic variance from CB_GWAS (Table S2). Within the 65 genomic regions, several candidate genes were found to be related to growth traits (Table 2).

Four candidate genes (*MDFIC*, *RPS12*, *PDE4D*, *AQP4*) were putatively linked to ADG and BW in pigs, which played supporting roles in the biological process of physical or muscular growth. As a myogenic regulatory factor, the post-transcription downregulation of *MDFIC* (MyoD family inhibitor domain containing) controls the promotion of myogenic differentiation, which is beneficial for skeletal muscle development in bovines [50]. The gene *MDFIC* was putatively linked to the birth weight of piglets [51] and the meat to fat ratio in F2 × cross pigs [52], and harbored two SNPs which were associated with ADG on SSC18:31.02~31.04 Mb. Ribosomal protein S12 (*RPS12*), mapped by two SNPs on SSC1:30.88~30.92 Mb, was examined to regulate the process of nucleic acid translation and physical growth in Drosophila by controlling *Xrp1* expressing levels [53]. Induced by fibroblast growth factor 1 (*FGF1*), phosphodiesterase 4D (*PDE4D*) was associated with growth and fertility impairment [54] and lipolysis suppression [55]. Assigned to the most significant SNP on SSC16:111.36 Mb for trait BW, aquaporin 4 (*AQP4*) was identified to widely express in the digestive tract of guinea pigs, and the mediation of gastric acid secretion in mice [56,57], which affected the physical growth in animals.

Seven candidate genes (*PNPLA2*, *DEAF1*, *PLCD3*, *ANKRD55*, *RORB*, *NDUFS4*, *ALDH8A1*) were found to potentially affect the trait of BFT in pigs, and are involved in pathways of fatty acid metabolism, adipogenesis and insulin signaling. Candidate gene *PNPLA2* (patatin like phospholipase domain containing 2), overlapping a genomic region with GV of 2.88% on SSC2:0.01~0.96 Mb, controls the initial step in triglyceride hydrolysis of long-chain fatty acid esters in adipocyte and non-adipocyte, and regulates adiposomes size [58]. Crespo-Piazuelo et al. identified that *PNPLA2* was embedded in a region on SSC2:0~12.76 Mb which was associated with the abundance of three fatty acids in backfat in pigs [59]. Gene *DEAF1* (deformed epidermal autoregulatory factor 1 homolog) mapped an identified variant on SSC2, is involved in fatty acid deposition, and regulated the expression of peripheral tissue antigen in lymph node stromal cells among diabetic mice and humans [52,60,61]. Phospholipase c delta 3 (*PLCD3*) played a key role in phosphatidylinositol catabolism by hydrolyzing phospholipids into fatty acids [62], and was linked to backfat thickness in Chinese native pigs [63] and intramuscular fat deposition in African Ankole cattle [64]. Assigned to a significant SNP on SSC16, *ANKRD55* (ankyrin repeat domain 55) showed a high correlation with insulin resistance and lipid metabolism, which may result in adiposity and have a risk of Type 2 Diabetes in humans [65,66]. With low expression in hyperglycemic donors, retinoid-related orphan receptor beta (*RORB*) positively regulated insulin secretion in pancreatic β cells [67] and acted as a regulator of osteogenesis [68]. It was closed to a variant on SSC1 and reported that backfat thickness showed a positive correlation with intramuscular fatness, which is a key factor affecting the flavor and juiciness of pork [69,70,71]. Gene *NDUFS4* (NADH: ubiquinone oxidoreductase subunit S4), harboring two identified markers on SSC16:23.93~23.97 Mb, was reported to highly express in high intramuscular fat in pigs [72], and *ALDH8A1* (aldehyde dehydrogenase 8 family member A1) was shown to be significantly associated with fatness in large white pigs [73].

The annotation of candidate genes supported that cellular processes in early stages may impact birth weight of piglets, and consequently affect many post-natal traits including growth performance and meat quality. For BTHWT in Yorkshire, three SNPs located in SSC14:135.03~135.18 Mb that were shared in two single-marker models completely coincided with the QTL identified in the birth weight of Yorkshire piglets [74]. Closed to these imprinted 13 kb regions, erythroid differentiation regulatory factor 1 (*EDRF1*) mediated organ development and histological differentiation and controlled erythroid development and differentiation in primitive cells or organs, such as fetal liver and fetal kidney [75]. The gene DEAD-box helicase 32 (*DHX32*), overlapping the two most prominent SNPs, has an ability to unwind DNA and RNA, and is involved in embryogenesis or cellular growth and division by changing RNA structure [76]. Geminin DNA Replication Inhibitor (*GMNN*), closed to the BTHWT-linked marker on SSC7:19.60 Mb, plays a crucial role in the S and G2 phases of the cell cycle. The gene *GMNN* is involved in the regulation of DNA replication and cell fate control during embryonic development [77]. Moreover, *ZNF300* (zinc finger protein 300) served as a zinc finger-domain protein regulating embryonic development and transcription. Gene *TAF4B* (TATA-box binding protein associated factor 4b) was essential for the maintenance of spermatogonia stem cells in mammals [78].

In our study, 48 SNPs related to the four traits were identified in the two populations. In each single-marker analysis, we found several significant SNPs that were in high linkage disequilibrium within a small region. For example, 13 ADG-associated SNPs on SSC18 from 31 Mb to 35.1 Mb were partitioned into four blocks with high pairwise LDs of 0.86, 0.58, 0.99 and 0.80. However, single-marker analysis only identified the association between a single SNP and traits of interest, neglecting the LD effect of adjoining SNPs [18,19]. Since *p*-value was used to test the statistical significance of SNPs [79], markers were likely to lack the power to reach the stringent threshold when association analysis came to small sample size test or low heritability of trait. Bayesian methods, which derived from the genomic estimation of breeding value [1,79,80], simultaneously estimated SNP effects without any significance testing. To take the genetic interaction effect into account, these models investigated the variation of genomic regions by fitting multiple SNPs. The Bayes C model used in this study assumed that most variants had a non-zero effect, which was consistent with MLM method, but only a small part of SNPs had zero effect [80]. From the comparison between the Bayes C model and single-marker regression analysis, most of the significant SNPs were embedded in the genomic regions with large variances, indicating that the linked SNPs did have large effects and confirming that the results of the two methods were in high accordance. On the other hand, numerous regions covered no significant SNP in single-marker analyses and also accounted for over 1% of genetic variations, such as the genomic region on SSC2:162.08~162.31 Mb explaining 6.11% of genetic variance with no identified SNP in CL_GWAS for BFT in Landrace. The single variant within the SSC2:162.08~162.31 Mb region was unable to surpass the significance threshold in CL_GWAS, but the total effects of these variants in this region might be substantial due to loci interactions. It has been reported that non-significant SNPs might have large effects for phenotype variability, and putatively causal variants that failed to surpass the threshold could be addressed by Bayesian analyses [81]. However, IL_GWAS identified a BFT-related locus (−log(*p*-value) > 8) within that region, suggesting that increased marker density might construct a more precise genomic relatedness matrix, and was able to highlight non-significant SNPs among genomic regions capturing large genetic variance. GWAS based on genomic regions with a Bayesian model was able to effectively discover putative QTLs and candidate genes [82,83], but to date few studies performed Bayesian GWAS on growth traits.

Performing GWAS with imputed genotypes is an effective way to enhance SNP density at a low cost [84]. We identified 142 loci associated with four traits across two breeds, composed of 122 novel loci and 20 common loci identified with CL_GWAS. With the IL_GWAS, some of the significant loci obtained a much stronger significance to surpass a more stringent threshold. For instance, the suggestive-significant marker CNC10110504 in CL_GWAS became the peak variant across the genome. A possible reason is that the overall effect of the genomic relatedness matrix in IL_GWAS was different from that in CL_GWAS [85,86]. However, the advantage of higher SNP density was not observed in Landrace.

### 3.4. Meta-Analyses

**Figure 4:**
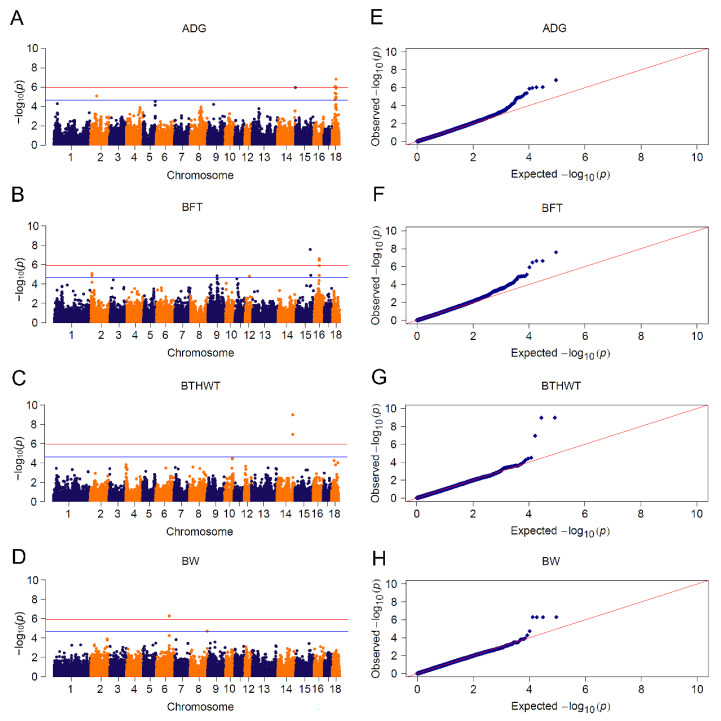
Meta-analyses for 4 growth traits. Manhattan plots for: (**A**) ADG: average daily gain; (**B**) BFT: backfat thickness; (**C**) BTHWT: birth weight and (**D**) BW: body weight. Q-Q plots for: (**E**) ADG, (**F**) BFT, (**G**) BTHWT and (**H**) BW. The red and blue lines in plots represent genome-wide and suggestive significance thresholds, respectively.

Meta-analysis uplifted the detection power by taking all informative SNPs into account, including those SNPs filtered out by quality control in single population GWAS [28]. We used meta-analysis to uncover potential associated loci by pooling CL_GWAS of Yorkshire and Landrace breeds. We detected 40 significant SNPs associated with four traits: fourteen for ADG, twelve for BFT, three for BTHWT and five for BW (Figure 4 and Table S4), 30 of which were consistent with CL_GWAS by single population. Among the ten novel SNPs (Table S4), three neighboring loci associated with ADG in SSC18:25.13~25.23 Mb were in the range of *PTPRZ1* (protein tyrosine phosphatase receptor type Z1), which encodes protein tyrosine phosphatase that regulates the cell proliferation on the embryonic spinal cord. Another ADG-associated SNP located in SSC15:0.17 Mb was closely adjacent to candidate gene signal transducing adaptor molecule 2 (*STAM2*) which plays a role in intracellular signal transduction induced by growth factors and cytokines [87]. Yang et al. detected seven genetic variants of the *STAM2* gene and demonstrated that these variants had significant associations with growth performance in Chinese Wuchuan Black cattle [88]. Moreover, two candidate genes, *ADAMTS6* (a disintegrin and metalloproteinase with thrombospondin motifs 6) and *BMP2K* (bone morphogenetic protein 2 inducible protein kinase), were assigned to the biological pathway of skeletal development and patterning. The lack of gene *ADAMTS6* leads to a drastic reduction in aggrecan and cartilage link protein, the impairment of bone morphogenetic proteins (BMP) signaling in cartilage and the detention of growth differentiation factors, which consequently result in impaired skeletal development [89]. BMP plays a key role in skeletal development and patterning, and protein encoded by *BMP2K* is considered as a kinase potentially regulating the attenuation of osteoblast differentiation [90]. Since body fatness was reported to negatively correlate with bone weight, bone mineral content and density [91], *ADAMTS6* and *BMP2K* may play roles in body weight in pigs.

### 3.5. Genomic Prediction

**Figure 5:**
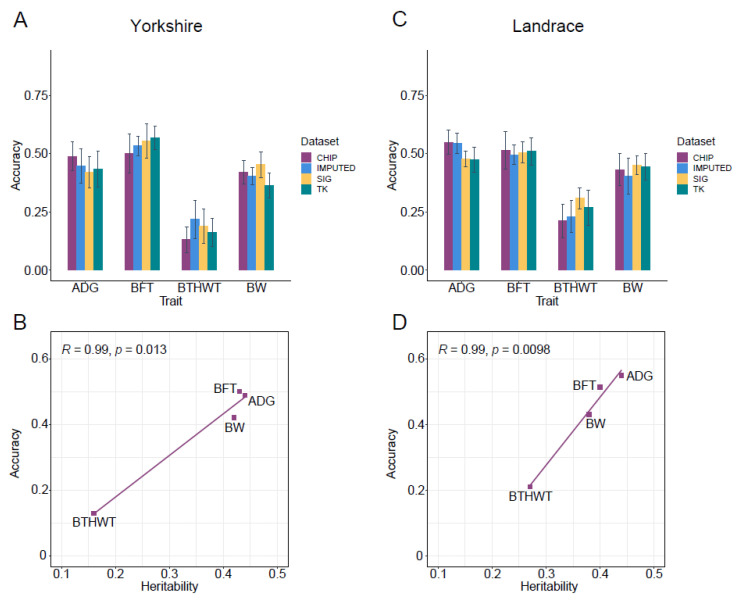
Genomic prediction accuracies (GPA) of GS using three sets of SNPs for average daily gain (ADG), backfat thickness (BFT), birth weight (BTHWT) and body weight (BW). Results for Yorkshire and Landrace are in (**A**,**C**), respectively. CHIP and IMPUTED represent GPAs estimated by GBLUP model based on all loci in 60K chip and imputed data, respectively. SIG represents GPAs estimated by GBLUP model based on the combination of significant variants from CL_GWAS, CB_GWAS and IL_GWAS. TK represents GPAs estimated by two-kernel based GBLUP based on the combination of significant variants from CL_GWAS, CB_GWAS and IL_GWAS. (**B**,**D**) represent correlation fitting curves between heritabilities and GPAs of 60K chip SNPs in Yorkshire and Landrace, respectively. Error bars represent the standard deviation of the accuracy across replications.

Genomic prediction using GBLUP and a 60K chip panel (CHIP) was set as a benchmark (Figure 5A,C). GPAs for three traits (ADG, BFT and BW) with moderate heritability ranged from 0.42 to 0.50 in Yorkshire, and from 0.43 to 0.55 in Landrace (Table S6). The prediction accuracies of the trait BTHWT with low heritability were 0.13 ± 0.05 and 0.21 ± 0.07 in the two breeds. The heritabilities and prediction accuracies based on the 60K chip panel for the four traits were highly positively correlated in the two breeds (r = 0.99) (Figure 5B,D).

Compared with using SNP chip data, the prediction accuracy using imputed WGS data had almost no improvement for ADG and BW in both breeds, which was contrary to the report for ADG in pigs [2], while for BTHWT prediction accuracies in Yorkshire and Landrace were increased by 22% and 10%, respectively. For the prediction accuracies of BFT, no improvement was observed in Landrace, which was similar to studies for backfat thickness in pigs reported by Zhang et al. [2] and Pérez-Enciso et al. [92], but a 7% improvement was yielded with the WGS dataset in Yorkshire. Genomic prediction improved with a large amount of variants identified for traits of BFT and BTHWT in Yorkshire, which was congruent with previous reports that genomic prediction with imputed data was affected by the genetic architecture of traits [2,5,92], although it can also be influenced by factors such as the statistical models used [92,93] and LD pruning before imputation [85,94].

We used significant variants (Table 2 and Table S4) preselected from CL_GWAS, IL_GWAS, meta-analysis, and SNPs located in genomic regions with high GV% from CB_GWAS in GS (SIG). In the comparison with using the complete chip data, prediction accuracies resulting from preselected SNPs for BFT in Yorkshire were 0.56 ± 0.08, which increased by 10%, and likewise for BW the GPAs increased by 8% and 4% in Yorkshire and Landrace, respectively (Figure 5A,C). The improvement was even more significant for the trait with low heritability, such as BTHWT, with GPA increasing by 45% in Yorkshire and 46% in Landrace. Taken together, the use of preselected SNPs had the potential to improve the GPA by reason of being free from the interference of non-significant loci [7,8]. Corredor et al. found that genomic prediction with SNPs identified to be linked to the vulva size in pigs had a better performance than that using all SNPs in the chip panel [11]. Van den Berg et al. suggested using only variants closed to the causal variants, and rare sequence variants closed to rare causal variants could improve prediction accuracies in dairy cattle, with accuracies ranging from 0.73 to 0.95 [95]. Such improvements were not observed in some studies which only used one of the GWAS methods to identify significant SNPs [96,97]. We then hypothesized that prediction accuracy using preselected data could be determined by the number of causal SNPs detected [98]. We pooled all SNPs identified by three different GWAS methods in genomic prediction. For example, significant causal variants missed by using a single GWAS method might be identified by other GWAS methods, which can be considered as supplements to the main model used, although there may be more false positive loci with no effects identified. For BFT in Yorkshire, GPAs estimated by two-kernel based GBLUP (TK) saw a slight improvement compared to SIG (Figure 5A,C). Compared with CHIP, TK improved by 23% and 29% for BTHWT in the two breeds, and by 4% for BW in Landrace. A similar study [9] revealed that TK outperformed the single kernel-based model with pre-selected variants by 0.11, and with all markers on the SNP panel by 0.06 in bulls, suggesting that the prioritization or appropriate weighting of pre-selected functional markers have advantages in GS for some traits.

## 4. Conclusions

In this study, we employed three different statistical methods as well as 60K SNP-chip and whole genome sequence data (CL_GWAS, CB_GWAS, IL_GWAS and meta-analysis based on chip data) for GWAS, which allowed us to discover more candidate loci linked to the four growth traits. A total of 1485 candidate loci, and 24 candidate genes which are involved in skeletal muscle development, fatty deposition, lipid metabolism and insulin resistance, were identified. Using the pre-selected functional SNPs in GS outperformed using all 60K SNP-chip and imputed WGS data for some traits, suggesting that the prioritization of preselected functional markers in GS models has a potential to improve prediction accuracies in livestock breeding.

## Figures and Tables

**Table 1:** Phenotypic statistics and SNP chip-based heritability estimations for four growth-related traits in two breeds.

Breed	Trait	N	Mean ± SD	CV	Max	Min	h^2^
Yorkshire	ADG	1734	565.32 ± 44.92	7.95	792.59	394.51	0.44
	BFT	1734	11.09 ± 3.34	30.12	28.04	5.17	0.43
	BTHWT	887	1.39 ± 0.25	17.99	2.42	0.60	0.16
	BW	1734	112.45 ± 14.64	13.02	167.10	74.01	0.42
Landrace	ADG	1123	608.19 ± 53.69	8.83	856.78	421.54	0.44
	BFT	1123	14.58 ± 4.88	33.47	34.95	5.46	0.40
	BTHWT	405	1.42 ± 0.26	18.31	2.17	0.71	0.27
	BW	1123	117.12 ± 16.25	13.87	160.12	80.09	0.38

ADG, average daily gain; BFT, backfat thickness; BTHWT, birth weight; BW, body weight.

**Table 2:** Summary of genomic regions significantly associated with growth-related traits of three statistical analyses.

Traits ^1^	Breeds ^2^	The Number of Significant SNPs			Candidate Genes
		CL_GWAS ^3^	CB_GWAS ^4^	IL_GWAS ^5^	
ADG	YY	14	242 (12)	6	*MDFIC*, *FOXP2*, *DOCK4*, *IMMP2L*, *ZPLD1*, *CYP7B1*
	LL	3	164 (7)	1	*ALDH8A1*, *RPS12*
BFT	YY	20	219 (11)	71	*UMAD1*, *GLCCI1*, *PDE4D*, *ZSWIM6*, *RNF180*, *ANKRD55*, *NDUFS4*, *NDUFA4*
	LL	5	193 (12)	2	*ODF3*, *DEAF1*, *PACS1*, *ZNF300*, *MS4A8*, *MS4A13*
BTHWT	YY	4		20	*EDRF1*, *DHX32*, *GMNN*, *MPP7*, *CUBN*, *ITGA8*, *RPP38*, *UCMA*
	LL		104 (5)		*ASAP1*, *NAV3*, *MROH5*, *PTP4A3*, *GPR20*
BW	YY	6	245 (12)	40	*TAF4B*, *AQP4*, *RORB*, *ATXN1*, *TAFA5*, *SELENOI*, *TMEM104*
	LL	1	174 (10)	2	*AMER2*, *MTMR6*, *NUP58*, *ATP8A2*, *SHISA2*, *FAM171A1*

^1^ ADG: average daily gain; BFT: backfat thickness; BTHWT: birth weight; BW: body weight. ^2^ YY: Yorkshire population; LL: Landrace population. ^3^ The number of significant SNPs identified in CL_GWAS with a threshold of 2.25 × 10^−5^. ^4^ The total number of SNPs located in genomic regions identified in CB_GWAS, explaining more than 1% of genetic variance. The digit in brackets represents the number of genomic regions identified in CB_GWAS, explaining more than 1% of genetic variance. ^5^ The number of significant SNPs identified in IL_GWAS with a threshold of 6.81 × 10^−7^.

## Data Availability

Data will be available upon request.

## Supplementary Materials

The following supporting information can be downloaded at: https://www.mdpi.com/article/10.3390/ani13040722/s1. Table S1: Significant SNPs related to growth traits by breed in CL_GWAS. Table S2: Genomic regions explained ≥ 1% genomic variance from CB_GWAS in traits by breed. Table S3: Significant SNPs related to growth traits by breed in IL_GWAS. Table S4: Significant SNPs identified in meta-analysis for four traits. Table S5: Sequencing depth of WGS data of reference panel. Table S6: Genomic prediction accuracies of different strategies by traits. Figure S1: Manhattan plots for BTHWT (birth weight) by breed in different GWAS approaches. Figure S2: Manhattan plots for BW (body weight) by breed in different GWAS approaches. Figure S3: Q-Q plots for CL_GWAS of four growth traits in Yorkshire and Landrace population. Figure S4: Q-Q plots for IL_GWAS of four growth traits in Yorkshire and Landrace population.
