# Supplementary material for: Utilizing Variants Identified with Multiple Genome-Wide Association Study Methods Optimizes Genomic Selection for Growth Traits in Pigs
Source: Animals (Basel). 2023 Feb 17;13(4):722. doi: 10.3390/ani13040722 (PMC9952664; doi:10.3390/ani13040722)
Supplement: Supplementary file 1 [file animals-13-00722-s001.zip › Supplementary File S1.pdf]

Supplementary file S1

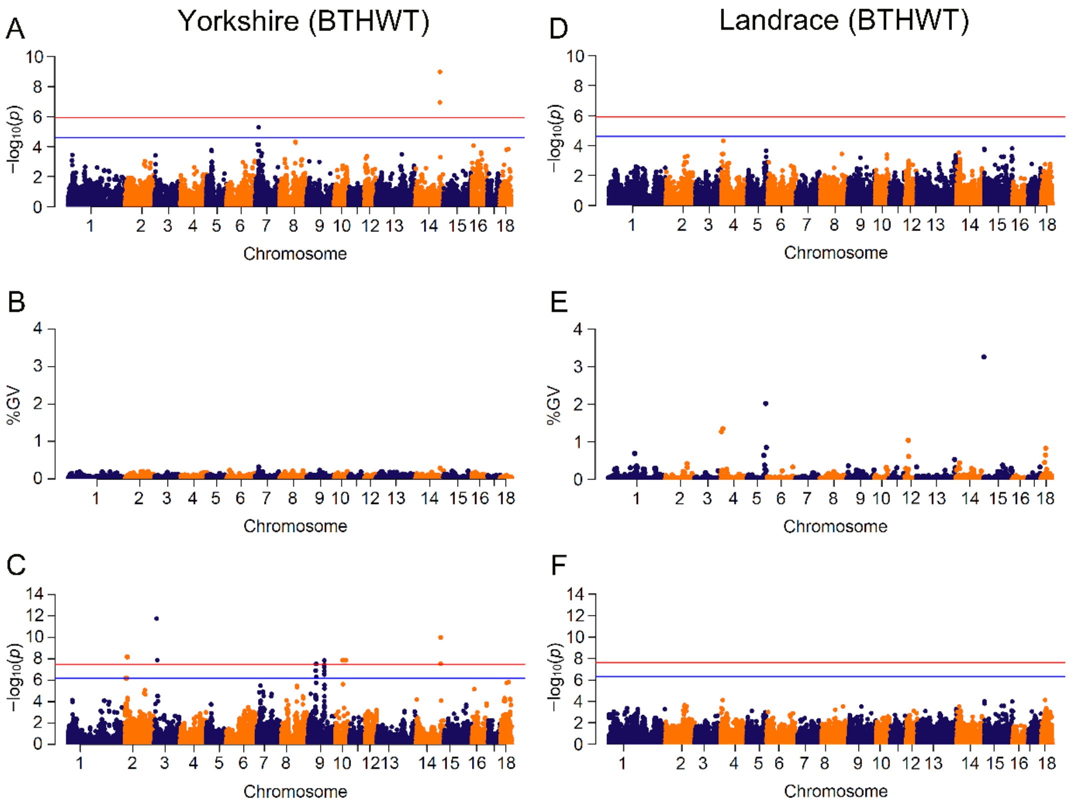

Figure S1. Manhattan plots for BTHWT (birth weight) by breed in different GWAS approaches. The red and blue lines in plots represent genome-wide and suggestive significance thresholds, respectively. Results in Yorkshire population for: (**A**) CL_GWAS; (**B**) CB_GWAS; (**C**) IL_GWAS. Results in Landrace population for: (**D**) CL_GWAS; (**E**) CB_GWAS; (**F**) IL_GWAS. %GV denotes the proportion of genomic variance explained by the region identified.

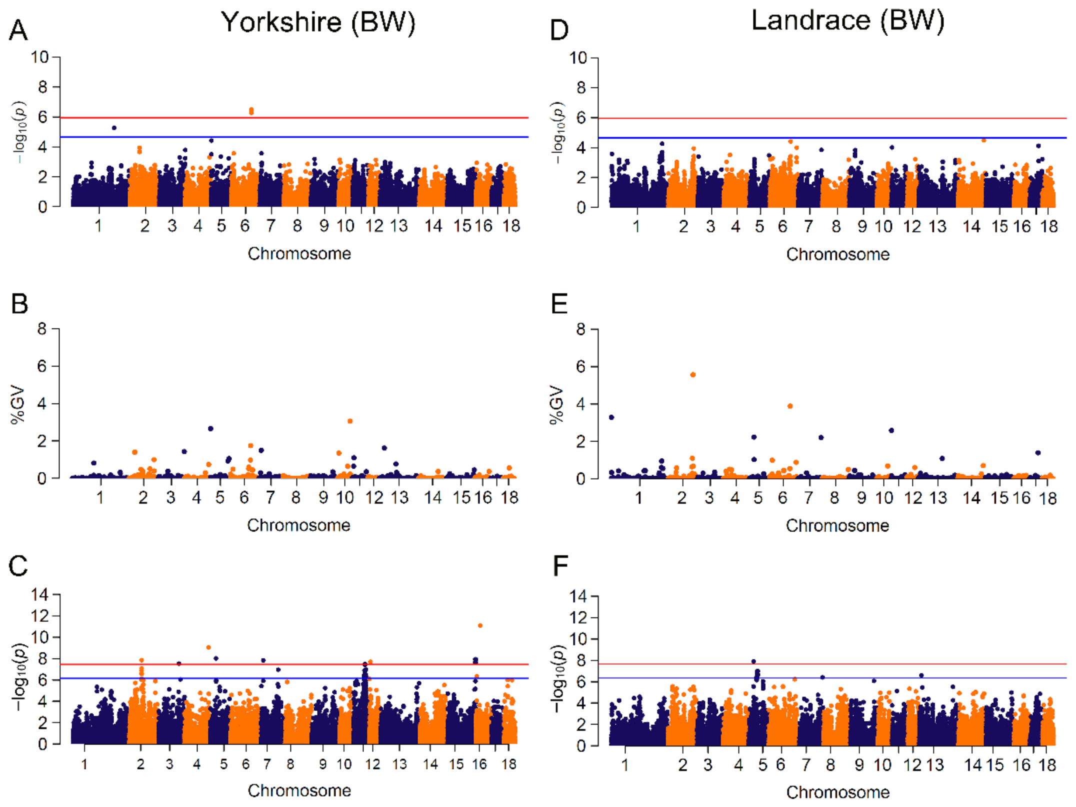

Figure S2. Manhattan plots for BW (body weight) by breed in different GWAS approaches. The red and blue lines in plots represent genome-wide and suggestive significance thresholds, respectively. Results in Yorkshire population for: (**A**) CL_GWAS; (**B**) CB_GWAS; (**C**) IL_GWAS. Results in Landrace population for: (**D**) CL_GWAS; (**E**) CB_GWAS; (**F**) IL_GWAS. %GV denotes the proportion of genomic variance explained by the region identified.

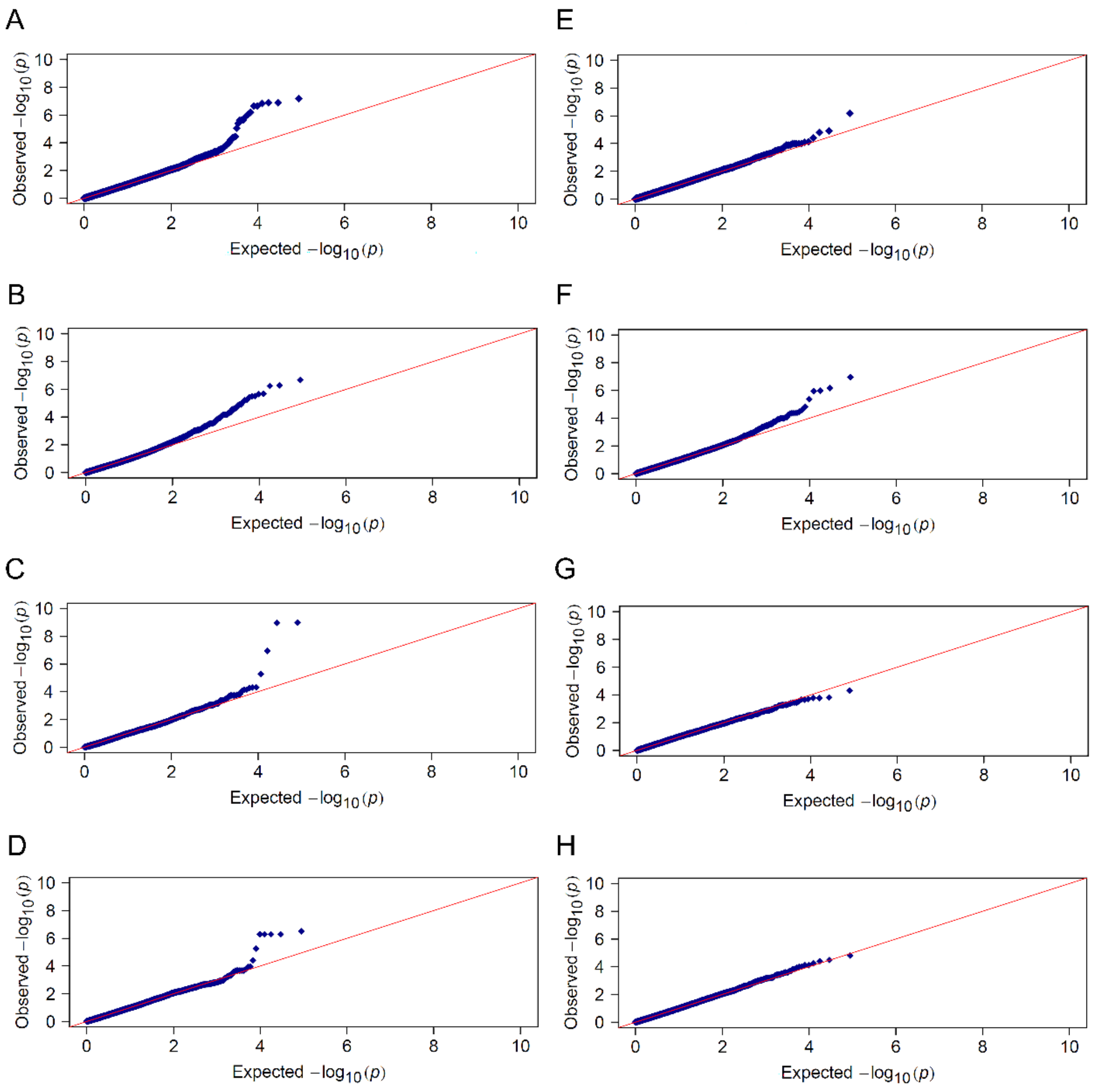

Figure S3. Q-Q plots for CL_GWAS of 4 growth traits in two breeds. Q-Q plots of Yorkshire population for: (**A**) ADG: average daily gain; (**B**) BFT: backfat thickness; (**C**) BTHWT: birth weight; (**D**) BW: body weight. Q-Q plots of Landrace population for: (**E**) ADG; (**F**) BFT; (**G**) BTHWT; (**H**) BW.

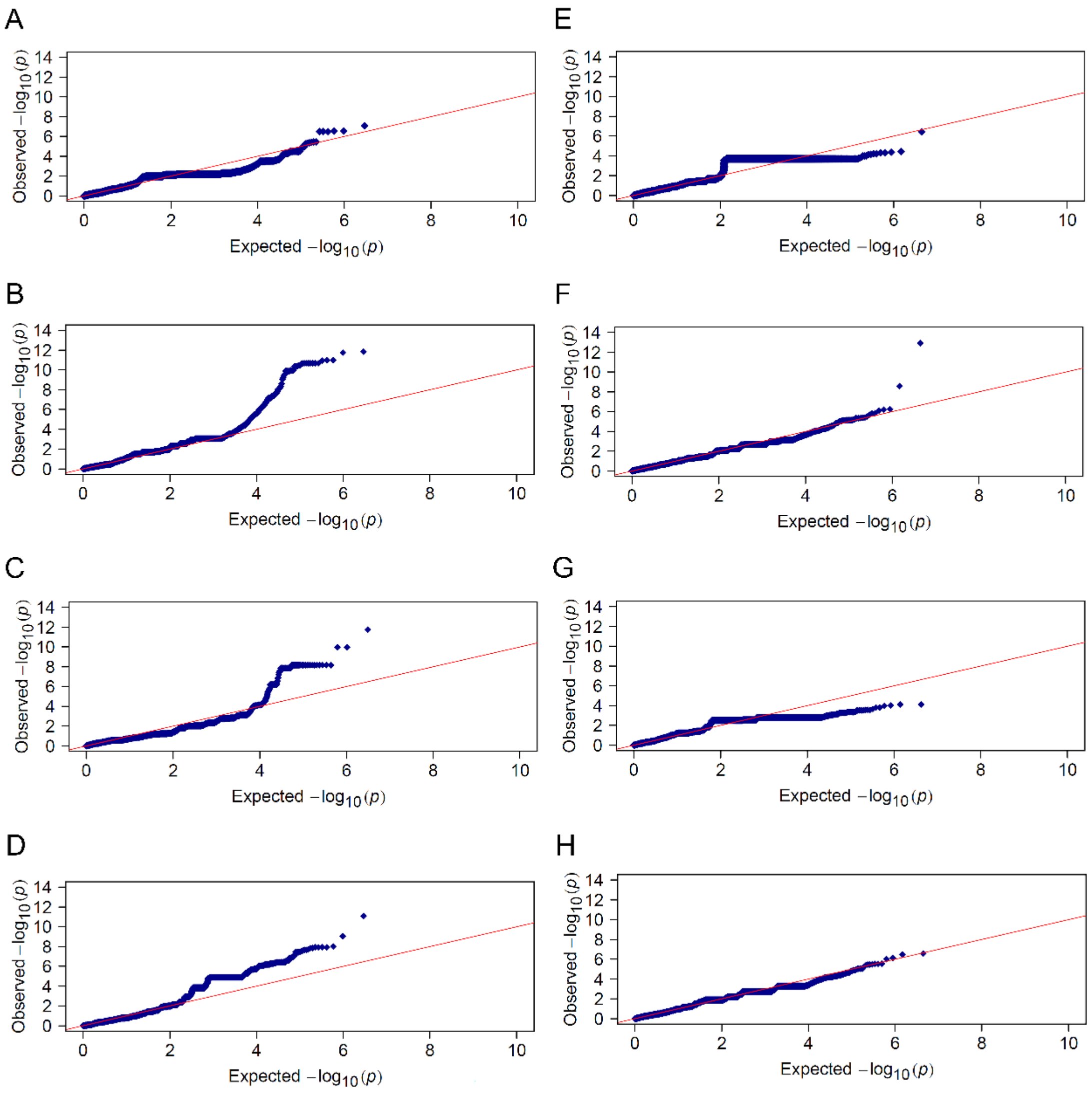

Figure S4. Q-Q plots for IL_GWAS of 4 growth traits in two breeds. Q-Q plots of Yorkshire population for: (**A**) ADG: average daily gain; (**B**) BFT: backfat thickness; (**C**) BTHWT: birth weight; (**D**) BW: body weight. Q-Q plots of Landrace population for: (**E**) ADG; (**F**) BFT; (**G**) BTHWT; (**H**) BW.
